# Supplementary material for: Increased Cuticle Waxes by Overexpression of WSD1 Improves Osmotic Stress Tolerance in Arabidopsis thaliana and Camelina sativa
Source: Int J Mol Sci. 2021 May 13;22(10):5173. doi: 10.3390/ijms22105173 (PMC8153268; doi:10.3390/ijms22105173)
Supplement: Supplementary file 1 [file ijms-22-05173-s001.zip › ijms-1181153-supplementary.pdf]

Supporting Information

Increased Cuticle Waxes by Overexpression of WSD1 Improves Osmotic Stress Tolerance in *Arabidopsis thaliana* and *Camelina Sativa*

Hesham M. Abdullah[1,2, ¥], Jessica Rodriguez[1], Jeffrey M. Salacup[3], Isla S. Castañeda[3], Danny J. Schnell[4], Ashwani Pareek[5], and Om Parkash Dhankher[1*]

Supplementary Figure S1

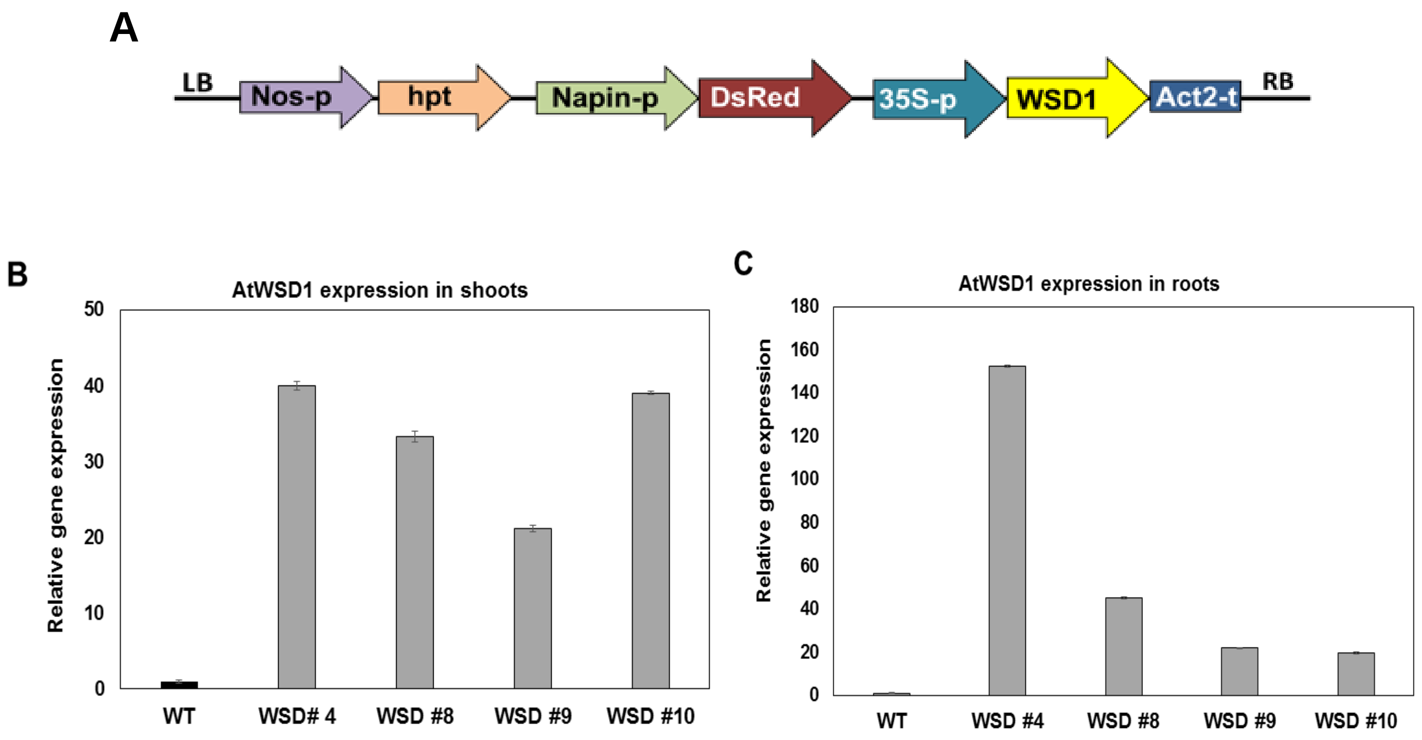

Supplementary Figure S1. Genetic transformation of *A. thaliana* and confirmation of *WSD1* gene expression into Arabidopsis shoots and roots. Simplified diagram illustrated the T-DNA construct designed to manipulate cuticular waxes via constitutive overexpression of WSD1 **(A)**; qRT-PCR analysis to detect the expression of *WSD1* in Arabidopsis shoots **(B)** and roots **(C)** in control WT and transgenic lines namely, WSD #4, 8, 9, and 10.

Supplementary Figure S2

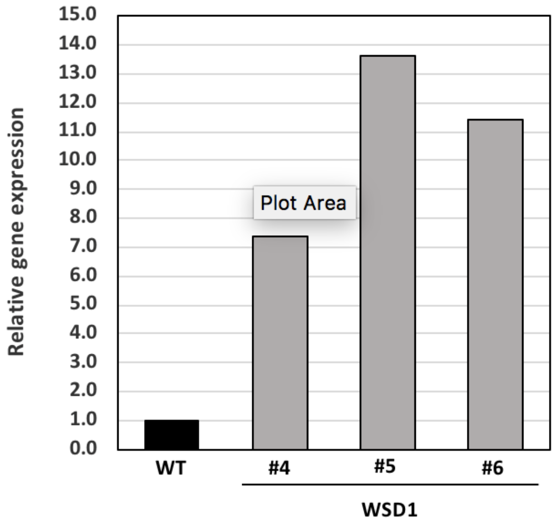

Supplementary Figure S2. qRT-PCR analysis to detect the expression of *WSD1* in *Camelina sativa* shoots in non-transgenic WT and transgenic lines WSD #4, 5, and 6.

Supplementary Figure S3

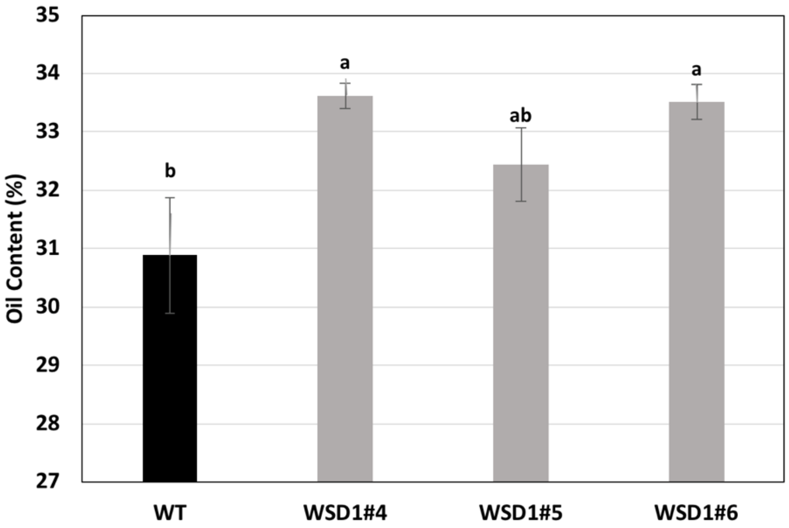

Supplementary Figure S3. The effect of WSD1 expression on total seed oil contents in Camelina transgenics. Total seed oil contents (%) of Camelina seeds overexpressing WSD1 (WSD #4, 5, and 6) and WT controls were analyzed. Data represents the mean of 4 independent plants of each lines, and the bars represent Standard error (±*SE*, *n=4*). Different letters denote significance of differences between WT and transgenic lines (Student's t-test) at $P < 0.01$ and $P < 0.05$.

Supplementary Figure S4

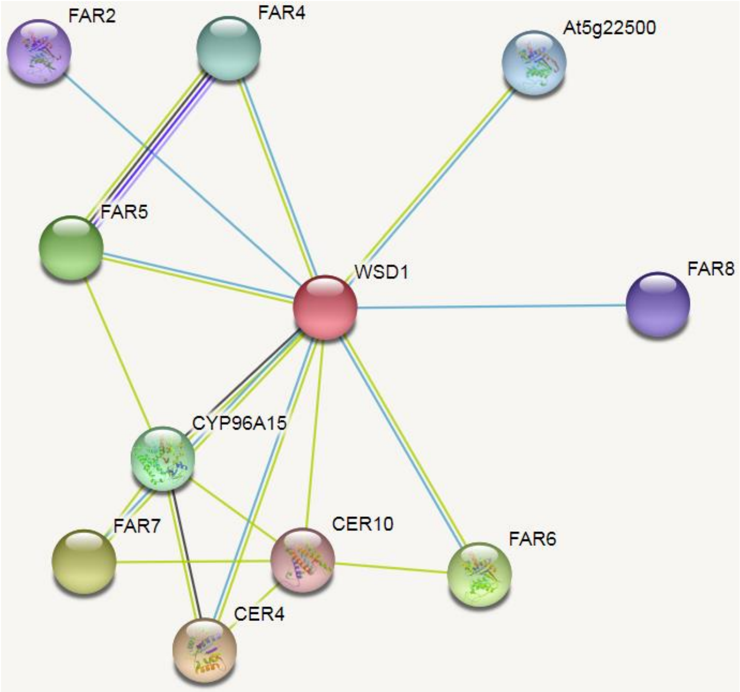

Edges:

Edges represent protein-protein associations

associations are meant to be specific and meaningful, i.e. proteins jointly contribute to a shared function; this does not necessarily mean they are physically binding each other.

Known Interactions

- *from curated databases*
- *experimentally determined*

Predicted Interactions

- *gene neighborhood*
- *gene fusions*
- *gene co-occurrence*

Others

- *textmining*
- *co-expression*
- *protein homology*

Your Input:

WSD1	*O-acyltransferase (WSD1-like) family protein; Encodes a bifunctional enzyme, wax ester synthase (WS) and diacylglycerol acyltransferase (DGAT). In vitro assay indicated a ratio of 10.9 between its WS and DGAT activities. Both mutant and in vivo expression/analysis in yeast studies indicated a role in wax biosynthesis; In the N-terminal section; belongs to the long-chain O-acyltransferase family (481 aa)*

Predicted Functional Partners:

		Neighborhood	Gene Fusion	Cooccurence	Coexpression	Experiments	Databases	Textmining	[Homology]	**Score**
CER4	*Jojoba acyl CoA reductase-related male sterility protein; Catalyzes the reduction of fatty acyl-CoA to fatty alcohols. The pr...*						●	●		0.989
FAR7	*Fatty acid reductase 7 (FAR7); Its function is described as oxidoreductase activity, acting on the CH-CH group of donors, bi...*						●	●		0.976
FAR6	*Fatty acyl-CoA reductase 6, chloroplastic; Catalyzes the reduction of fatty acyl-CoA to fatty alcohols. May be involved in th...*						●	●		0.968
FAR5	*Probable fatty acyl-CoA reductase 5; Catalyzes the reduction of fatty acyl-CoA to fatty alcohols. Catalyzes specifically the f...*						●	●		0.944
CYP96A15	*Cytochrome P450, family 96, subfamily A, polypeptide 15; Involved in the formation of secondary alcohols and ketones in s...*				●			●		0.932
FAR4	*Probable fatty acyl-CoA reductase 4; Catalyzes the reduction of fatty acyl-CoA to fatty alcohols. Catalyzes specifically the f...*						●	●		0.924
At5g22500	*Fatty acyl-CoA reductase 1; Catalyzes the reduction of fatty acyl-CoA to fatty alcohols. Catalyzes specifically the formation...*						●	●		0.904
FAR8	*Fatty acyl-CoA reductase 8; Catalyzes the reduction of fatty acyl-CoA to fatty alcohols. Catalyzes specifically the formation...*						●			0.900
FAR2	*Jojoba acyl CoA reductase-related male sterility protein; Catalyzes the reduction of fatty acyl-CoA to fatty alcohols. Involve...*						●			0.900
CER10	*3-oxo-5-alpha-steroid 4-dehydrogenase family protein; Catalyzes the last of the four reactions of the long- chain fatty acids...*							●		0.898

Supplementary Figure S4. *In-silico* protein-protein interaction analysis by seraching the STRING database (https://string-db.org/) to identify any known protein(s) that may physically or functionally interact with WSD1.

Supplementary Materials and Methods

Generation of Arabidopsis and Camelina transgenics and growth conditions

The *Arabidopsis thaliana* (ecotype: Columbia) and *Camelina sativa* (cultivar: Suneson) transgenic lines were generated by introducing the Arabidopsis *WSD1* gene using the gene cassette *CaMV35S-p:WSD1:Actin2-t* as shown in supplemental Fig. S1. This cassette was cloned into the plant expression vector modified *pCAMBIA-DsRed* (Fig. S1) and was used to transform Arabidopsis and Camelina using *Agrobacterium*-mediated flower-dip method according to [36] using a protocol modified from [41, 42]. Arabidopsis and Camelina plants at maturity were harvested. The T1 seeds of Arabidopsis and Camelina were selected for the expression of DsRed fluorescence and/or for hygromycin antibiotic resistance on hygromycin-containing medium.

The growth conditions for Arabidopsis plants were applied as described in (Dixit et al., 2018), plants were grown in controlled environment growth chamber with 16/8 hr light/dark photoperiods and 22/18 ℃ day/night temperatures. The growth conditions for Camelina plants were applied as described in [35, 36]. The seeds were germinated, and seedlings were maintained in the greenhouse room at 22 ℃ under natural light conditions supplemented with high-pressure sodium lights (light threshold of 566 μmol/m2/s) with a 16-h photoperiod (16 h of light and 8 h of darkness), and a 50% minimum humidity. Plants were watered regularly and were fertilized with 200 ppm N of Peters Professional 20-10-20 Peat-lite water-soluble fertilizer.

PCR genotyping and qRT-PCR for gene expression analysis

To measure the expression level of the *WSD1* gene in the transgenic and WT plants, total RNA was isolated from leaf tissue using the RNeasy mini kit (Sigma-Aldrich), and then cDNA was synthesized using Verso cDNA synthesis kit (Thermo Scientific). The qRT-PCR was performed following the instructions for the Mastercycler ep realplex (Eppendorf) using gene-specific primers with SYBR green mix kit (Thermo Fisher Scientific) according to the manufacturer's protocol. Gene expression levels were normalized with respect to the internal control *elongation factor 1α* (*EF1α*). Three biological and three technical replicates of each sample were used for qRT-PCR analysis. The mean ± SE levels of relative abundance obtained from three independent measurements are reported.

Differential regulation of *AtWSD1* under abiotic stress treatments

Wild type Arabidopsis, ecotype Columbia, seeds were surface-sterilized as described in [43]. The sterilized seeds were germinated and grown on a nylon screen placed on top of ½ strength solid MS media with vitamins (PhytoTechnology Laboratories, KS, USA), supplemented with 0.8% (w/v) Phytoblend agar (Caisson Laboratories, UT, USA), and 1% (w/v) sucrose, in Petri dishes for 10 days under the growth conditions mentioned above. Seedlings grown on nylon screen were then transferred to a 2 cm long piece of glass tube support placed in the magenta boxes containing ½ strength liquid MS media for additional 8 days before exposure to 100 mM D-mannitol, 100 mM NaCl, and 2.5 μM ABA in liquid culture. The abiotic stress response in plants was induced by replacing the ½ strength MS media for each treatment with the above-mentioned stress agents. Plant shoot and root samples were harvested separately at 0, 6, 12, and 24 hr time intervals; washed with deionized water, flash frozen in liquid nitrogen, and stored at −80 ℃ until RNA extraction and qRT-PCR analyses. For each treatment, three biological replicates and three technical replicates were obtained.

Plant growth assays for mannitol, ABA, drought, and salt tolerance

Stress tolerance/sensitivity assays were performed as described previously [42]. The WT and WSD1 transgenic seeds were germinated in ½ strength MS agar plates (with 1% sucrose) in the presence or absence ABA (1 uM), D-mannitol (100 mM), and sodium chloride (NaCl, 75 mM) and the seedlings were maintained for 21 days with a 16/ 8 hr light/dark cycle at 22 /18 ℃ day/night temperature. To reduce variations, 12 seeds each of the wild type and transgenic lines were grown side by side on the same plate, and their growth was compared. For each stress treatment, four replicates of each transgenic line along with wild type control plants were grown. At the end of ~ 21 days, plants were photographed, shoot fresh weight and root length was recorded. The experiment was repeated independently in three sets, and the results were consistent.

For drought stress, equal weights of potting mix soil were transferred to pots before transplanting the Arabidopsis plants. While the plants were growing, each pot received equal volumes of water. After attaining sufficient biomass (4-wk-old seedling), the pots were saturated with tap water before starting the drought treatment by water withholding. Watering was withheld for 12 days until plants showed signs of wilting. The soil moisture was recorded daily during this period. Subsequently, the plants were re-watered to recover from stress and were analyzed for signs of permanent damage and photographed. For salt stress, ~ 28 days old plants grown in 4.0-

inch pots were saturated with either tap water to serve as the control or with 100 mM NaCl solution in trays for 6 days. Following the 6-day treatment, the pots were placed in trays filled with tap water for several days to leach out the remaining salts from soil for recovery. For both salt and drought stress, at least five individual pots with five plants each were used, and the experiments were repeated.

For drought stress in Camelina, seeds were germinated in potting mix soil and then 2-weeks old seedlings were transferred to sand pots (5-inch width and 16-inch length) filled with an equal weight of sand. While the plants were growing, each pot received equal volumes of water, with fertilizers whenever needed. After attaining sufficient biomass, the pots were saturated with tap water before the drought treatment was applied by water withholding. The 5-week-old plants were then exposed to 50% and 75% reduction in soil moisture and soil moisture was recorded daily during this period. The signs of wilting were recorded in both WT and WSD1 expressing plants after 2 and 4 weeks of treatment. Plants were analyzed for signs of permanent damage such as dead leaves, yellowing of leaves, and drooping of branches and tip death and photographed. Under control (well-watered) and drought conditions, the fresh biomass and the number of branches in Camelina plants were recorded, and the plants were maintained till maturation. The seeds were harvested when plants matured and the seed pods were dry. Seeds harvested from each plant were weighed to determine the seed yield. The weight of 100 Camelina seeds was used to indicate the seed mass. For total plant biomass measurements, the shoots and roots, after seed harvesting, were dried at 70 ℃ for 4 days before weighing.

Cuticular wax loading and composition analysis

Cuticular waxes were extracted from Arabidopsis leaves (~500 mg) and stems (~200 mg) of 4-week old plants in chloroform (~5ml) for 30 seconds at room temperature following the method described by [30]. The extracted chloroform solvents were then evaporated under N_2 steam, and subsequently, the alkane standard (Heptatriacontane n-37, $C_{37}H_{76}$) was added as an internal standard for quantification purposes. The lipid extracts were redissolved in a mixture of 25 µl acetonitrile and 25 µl of bis-N,N-trimethylsilyltrifluoroacetamide (BSTFA) and then heated at 70 ℃ for 30 minutes to convert waxes into trimethylsilyl derivatives. The qualitative and quantitative analysis of cuticular waxes was conducted using gas chromatography with mass spectrometry (GC/MS) for identification, followed by gas chromatography with flame ionization

detection (GC-FID) for quantification. GC-FID was performed on Agilent 7890 equipped with a HP-5 column (60m, 0.32 mm ID, 0.25 um film) using hydrogen (H) as a carrier gas. The inlet temperature was 250°C. The oven program started at 70°C and then increased at 17◦C min^{-1} to 130◦C and subsequently increased at 7°C min^{-1} to 320°C. The final temperature was held for 30 min. GC/MS was performed on Agilent 6890 coupled to a 5973N single quadrupole mass spectrometer. The column, oven program, and inlet temperature were the same as for the GC-FID. Helium (He) was used as carrier gas. The GC-MS was run in full scan mode between 50 and 550 amu.

Chlorophyll Leaching Assays

The chlorophyll content in Arabidopsis leaves was determined as described in [30]. Briefly, detached leaves of 4-week old plants grown in soil under growth chamber conditions were used. Approximately 300 mg of each leaf sample were incubated in ice for 30 min and then immersed in 30 ml of 80% ethanol in 50 ml conical tubes at room temperature. At the indicated time points after the initial immersion, ~ 100 μl aliquots were removed from the solution, and the amount of extracted chlorophylls were quantified by measuring absorbance at 647 and 664 nm using an Evolution 60S UV-visible-spectrophotometer (Thermo Scientific). The amount of chlorophyll A, B and total chlorophyll was determined according to the formula described by (Arnon DI, 1949) of which ChlA (μg/ml)= 12.7 (A663)-2.69 (A645); ChlB (μg/ml)= 22.9 (A645)-4.68 (A663); total Chl (μg/ml)= 20.2 (A645)+ 8.02 (A663), respectively. Three measurements were used, and the average ± standard error (SE) of the data was indicated on the graphs.

Water loss assays

Four weeks old Arabidopsis plants grown in soil under growth chamber conditions were used for the leaf water loss assay following the methods described in [30]. Plants were dark-acclimated for ~10 hours, and then the detached rosette leaves were soaked in water for 60 min in the dark. The leaves were air-dried and weighed at the indicated time points, and the amount of water loss was presented as the percentage of the leaf weight at the indicated time points relative to the initial fresh weight. A minimum of three measurements was used, and the average ± standard error (SE) of the data was indicated on the graph.

Scanning electron microscopy (SEM)

Cryogenic SEM (ZEISS inc.) was used to view epicuticular wax crystallization patterns. Inflorescence stem segments from tip to 3 cm and the fourth rosette leaves were collected from Arabidopsis wild-type (Col-0) and WSD1 overexpressing plants after 6 weeks of growth as described previously [30, 45]. Samples were fixed in 5% glutaraldehyde in 0.1 M phosphate buffer (pH 6.8) for 2 hours at 4 ℃ before they washed in phosphate buffer three times for 10 minutes each. The samples were subsequently dehydrated in diluted series of ethanol (35%, 50%, 75%, 95%, 100%, 100%, 100%) for 15 minutes each. Then the dehydrated samples were immersed in 1-2 ml of 50% and then 100% hexamethyldisilazane (HMDS) for 10 minutes each before they were air-dried in a desiccator overnight at room temperature. The air-dried samples were adhered to the cryo-holder using carbon tape, and cryoadhesive were sputter-coated with platinum and then transferred to the microscope cryo-stage for imaging.

References

30. Seo, P.J.; Lee, S.B.; Suh, M.C.; Park, M.J.; Go, Y.S.; Park, C.M. The MYB96 transcription factor regulates cuticular wax biosynthesis under drought conditions in Arabidopsis. *Plant Cell* **2011**, 23, 1138-1152.

35. Abdullah, H.M.; Akbari, P.; Paulose, B.; Schnell, D.J.; Qi W.; Park, Y.; Pareek, A.; Dhankher, O.P. Transcriptome profiling of Camelina sativa to identify genes involved in triacylglycerol biosynthesis and accumulation in the developing seeds. *Biotechnology for Biofuels* **2016**, 9:136.

36. Chhikara, S.; Abdullah, H.M.; Akbari, P.; Schnell, D.J.; Dhankher, O.P. Engineering Camelina sativa (L.) Crantz for enhanced oil and seed yields by combining diacylglycerol acyltransferase1 and glycerol-3-phosphate dehydrogenase expression. *Plant Biotechnology* **2018**, 16(5), 1034-1045.

41. Lu, C.; Kang, J. Generation of transgenic plants of a potential oilseed crop Camelina sativa by Agrobacterium-mediated transformation. Plant Cell Reports **2008**, 27(2), 273-278.

42. Clough, S.J.; Bent, A.F. Floral dip: a simplified method for Agrobacterium-mediated transformation of Arabidopsis thaliana. *Plant Journal* **1998**, 16, 735-743.
43. Dixit, A.R.; Dhankher, O.P. A novel stress-associated protein “AtSAP10” from Arabidopsis thaliana confers tolerance to nickel, manganese, zinc, and high temperature stress. *PLoS ONE* **2011,** 6(6), e20921.
45. Lü, S.; Song, T.; Kosma, D.K.; Parsons, E.P.; Rowland, O.; Jenks, M.A. Arabidopsis CER8 encodes LONG-CHAIN ACYL-COA SYNTHETASE 1 (LACS1) that has overlapping functions with LACS2 in plant wax and cutin synthesis. *Plant Journal* **2009**, 59, 553-564.
